# Effects of predicted Khamisiyah exposure on default mode network resting state functional connectivity in Gulf War Veterans

**DOI:** 10.3389/ftox.2026.1772515

**Published:** 2026-03-05

**Authors:** Linda L. Chao, Salvatore Torrisi

**Affiliations:** 1 VA Advanced Imaging Research Center, San Francisco Veterans Affairs Healthcare Center, San Francisco, CA, United States; 2 Department of Radiology and Biomedical Imaging, University of California, San Francisco, San Francisco, CA, United States; 3 Department of Psychiatry and Behavioral Science, University of California, San Francisco, San Francisco, CA, United States

**Keywords:** attention, default mode network, Gulf War, nerve agents, neuroimaging, resting-state fMRI

## Abstract

**Introduction:**

Potentially more than 100,000 US troops were exposed to organophosphorus (OP) nerve agents when an ammunition bunker at Khamisiyah, Iraq was destroyed shortly after the end of the 1991 Gulf War (GW). We previously reported evidence of differences in brain structure and function in GW veterans with predicted exposure to the Khamisiyah plume compared to veterans without predicted exposure. Here, we investigate the effects of predicted exposure to the Khamisiyah plume on brain functional connectivity in the default mode network (DMN).

**Methods:**

Forty-one GW veterans (19 with and 22 without predicted exposure) underwent structural and resting-state magnetic resonance imaging (MRI) on a 3 Tesla scanner. Differences in DMN connectivity between veterans with and without predicted Khamisiyah exposure were examined using a left posterior cingulate cortex (PCC) seed-based analysis in AFNI. FreeSurfer was used to derive quantitative estimates of total hippocampal volume. The veterans were also assessed with the Conners Continuous Performance Test (CPT).

**Results:**

Compared to veterans without predicted exposure, those with predicted Khamisiyah exposure demonstrated weaker connectivity between the left PCC and a cluster in the caudal right anterior cingulate cortex (ACC). Veterans with predicted exposure also had smaller left hippocampal volume compared to unexposed veterans.

**Discussion:**

Although the cross-sectional nature of this study precludes conclusions about causality, the finding of decreased DMN functional connectivity in GW veterans with predicted Khamisiyah exposure warrants replication in a larger, independent sample. If confirmed, this result would add to the literature suggesting persistent differences in brain function between deployed GW veterans with and without predicted Khamisiyah exposure and argue for further investigation into the long-term effects of GW-deployment related exposures.

## 1 Introduction

Shortly after the end of the 1990-91 Gulf War (GW), the U.S. Army Corp of Engineers destroyed an underground ammunition bunker in Khamisiyah, Iraq. Later, it was discovered that the bunker contained rockets filled with organophosphorus (OP) nerve agents (Winkenwerder, 2002). The demolition operation exposed potentially more than 100,000 U.S. military personnel whose units were downwind from Khamisiyah to low levels of OP nerve agents (Directorate for Deployment Health Support of the Special Assistant to the Medical Readiness and Military Deployments, April 2002). Despite initial skepticism about assumptions made by the Department of Defense (DOD) and the Central Intelligence Agency’s (CIA) efforts to model the plume that resulted from the Khamisiyah demolition operation (US General Accounting Office, 2004), we and others have reported evidence of reduced gray and white matter (Chao et al., 2011; Chao et al., 2010; Heaton et al., 2007) and hippocampal (Chao et al., 2014; Chao et al., 2017; Chao et al., 2010) volume, disruptions in white matter microstructure (Chao and Zhang, 2018; Chao et al., 2015), and cognitive dysfunction (Chao et al., 2011; Proctor et al., 2006) in veterans with predicted Khamisiyah exposure compared to deployed GW veterans without predicted exposure. Our behavioral finding of increased omission errors on the Continuous Performance Test (CPT) (i.e., evidence of inattention, Chao et al., 2011) in GW veterans with predicted exposure mirrors the case report of a U.S. Army sergeant who was exposed to sarin in Iraq in 2004 when the improvised explosive device (IED) that he was disarming exploded (Loh et al., 2010). Eight months after his exposure, which was confirmed through depressed erythrocyte cholinesterase activity measured shortly after the IED explosion, the patient exhibited poor focused and divided attention on neuropsychological assessment (Loh et al., 2010).

Neurological and psychiatric patients who also exhibit poor focused and divided attention, such as individuals with attention deficit hyperactivity disorder (ADHD) (Castellanos et al., 2007; Sun et al., 2012; Tian et al., 2006; Zang et al., 2007) and Alzheimer’s disease (AD) (Dennis and Thompson, 2014; Greicius et al., 2004) have disruptions in the default mode network (DMN). The DMN is an intrinsic brain network comprised of the medial prefrontal and posterior cingulate cortices, precuneus, and lateral inferior parietal cortex (Fox and Raichle, 2007; Greicius and Menon, 2004). The term “default” comes from the observation that the DMN is most active when individuals are at rest, suggesting that our brains default to using this network when there is no externally directed task (Fox and Raichle, 2007). However, research has revealed that the DMN is also active during tasks that rely on internally generated representations such as remembering, envisioning the future, and making social inferences (Buckner and Carroll, 2007; Hassabis and Maguire, 2009; Spreng et al., 2009).

A few neuroimaging studies have investigated brain functional connectivity in GW veterans. One study of veterans with Gulf War Illness (GWI), the chronic multisymptom illness associated with military service during the Gulf War (Research Advisory Committee on Gulf War Veterans' Illnesses, 2008), and comorbid chronic fatigue syndrome (CFS) found significant task-related deactivations in the DMN following physical activity that induced post-exertional malaise in veterans with GWI and CFS (Rayhan et al., 2019). However, because the authors did not acquire continuous resting state fMRI (rs-fMRI) scans (i.e., no task; DMN activity in the study was derived by subtracting the control condition from an experimental N-back task condition), it was unclear if veterans with GWI and CFS had altered DMN activity at baseline prior to the exercise challenge. Another study that did acquire rs-fMRI scans in GW veterans reported impaired functional connectivity between language networks and sensory input networks of all modalities and motor output networks in veterans with GWI, defined according to the Haley Syndromes (Haley et al., 1997), Centers for Disease Control and Prevention Chronic Multisymptom Illness (CDC CMI) criteria (Fukuda et al., 1998), and Kansas GWI criteria (Steele, 2000) compared to veterans without GWI (Gopinath et al., 2019). There were no GWI-related differences in functional connectivity in the DMN or other networks associated with executive function, memory, or affective domains. However, Gopinath et al. (2019) speculated that this may have been due to the spatiotemporal separability constraints inherent in the independent component analysis approach that they employed. Recently, the same group used rs-fMRI connectomics and machine learning techniques to probe brain functional connectivity in veterans with GWI. This time, they found heterogenous functional connectivity impairments in veterans with different Haley syndromes (Yang et al., 2024). Specifically, the sensory thalamus node was the strongest hub of functional connectivity impairment in ill GW veterans with Haley Syndrome 1. The perirhinal and entorhinal cortices were the strongest hub of functional connectivity impairment in ill GW veterans with Haley Syndrome 2. The ventral caudate nucleus was the strongest hub of impairment in ill GW veterans with Haley Syndrome 3. Notably, there was an absence of brain functional connectivity difference when all three Haley syndrome groups were combined (i.e., CDC CMI cases or Kansas GWI cases) and compared with veterans without GWI symptoms.

To our knowledge, no study has examined brain functional connectivity in GW veterans with predicted exposure to the Khamisiyah plume. The study’s primary outcome was DMN functional connectivity. Because we previously found evidence of inattention in GW veterans with predicted exposure to the Khamisiyah plume (Chao et al., 2011), and because there have been reports of abnormal DMN functional connectivity in adults (Castellanos et al., 2007) and children (Sun et al., 2012) with ADHD, we hypothesize there would be DMN functional connectivity differences between GW veterans with and without predicted exposure to the Khamisiyah plume. Because we previously found hippocampal atrophy (Chao et al., 2014; Chao et al., 2017; Chao et al., 2010) and more errors of omission on the CPT (Chao et al., 2011) in GW veterans with predicted Khamisiyah exposure compared to unexposed GW veterans, we examined hippocampal volume and CPT performance exposure as secondary outcome measures.

## 2 Methods

### 2.1 Participants

Participants included 41 deployed GW veterans (34 males; 7 females) who took part in a larger study funded by the Department of Veterans Affairs that investigated the effects of predicted exposure to the Khamisiyah plume on brain structure and function. Demographic, military, and clinical characteristics of the 202 deployed GW veterans who took part in the parent study has been described elsewhere (Chao, 2018). Near the end of the parent study, we modified the study protocol to collect pilot resting state fMRI (rs-fMRI) data from the subset of remaining participants (n = 41). Nineteen (15 males; 4 females) veterans with pilot rs-fMRI data had predicted exposure to the Khamisiyah plume according to DOD and CIA modeling efforts. Twenty-two veterans (19 males; 3 females) veterans with pilot rs-fMRI data did not have predicted Khamisiyah exposure. One veteran’s (a 57-year-old male without predicted exposure) rs-fMRI data was excluded from analysis due to excessive head motion (0.5 mm Euclidean distance for per-volume censoring and >20% total run volumes censored).

Table 1 summarizes the demographic and clinical characteristics of the study sample. All participants signed informed consent forms approved by the Institutional Review Boards of the University of California, San Francisco and the San Francisco Veterans Affairs Healthcare System (SFVAHCS).

**TABLE 1:** Demographic and clinical characteristics of study sample.

Variable	Exposed (N=19)	Unexposed (N=22)	Statistics
Age (years)	55.7 (8.4)	55.8 (8.2)	*t* = 0.05, *df* = 39, *p* = 0.96
Education (years)	16.3 (2.6)	15.6 (2.5)	*t* = −0.96, *df* = 39, *p* = 0.34
No. (%) female	4 (21%)	3 (14%)	χ^2^ = 0.40, *df* = 1, *p* = 0.53
No. (%) Kansas GWI cases	10 (52.6%)	8 (36.4%)	χ^2^ = 3.27, *df* = 1, *p* = 0.07
No. (%) Kansas exclusionary condition(s)	5 (26.3%)	2 (9.1%)	χ^2^ = 2.14, *df* = 1, *p* = 0.14
No. (%) CDC CMI cases	17 (89.5%)	15 (68.2%)	χ^2^ = 2.70, *df* = 1, *p* = 0.10
Military history during the Gulf war			
Rank	​	​	χ^2^ = 0.02, *df* = 1, *p* = 0.90
Enlisted	15 (78.9%)	17 (77.3%)	​
Officer	4 (21.1%)	5 (22.7%)	​
Branch of service	​	​	χ^2^ = 20.43, *df* = 3, *p* < 0.001
Army	19 (100%)	7 (31.8%)	​
Air force	​	5 (22.7%)	​
Marines	​	6 (27.3%)	​
Navy	​	4 (18.2%)	​
Component of service	​	​	χ^2^ = 1.69, *df* = 2, *p =* 0.43
Active duty	14 (72.2%)	19 (85.7%)	​
Reserves	4 (22.2%)	3 (14.3%)	​
National Guard	1 (5.6%)	​	​
No. Possible/probable mild-moderate TBI	9 (47.4%)	9 (40.9%)	χ^2^ = 0.17, *df* = 1, *p =* 0.68
No. current PTSD	5 (26.3%)	2 (9.1%)	χ^2^ = 2.14, *df* = 1, *p =* 0.14
No. current MDD	3 (15.8%)	3 (13.6%)	χ^2^ = 0.04, *df* = 1, *p =* 0.85
No. history of alcohol dependence/abuse	7 (36.8%)	5 (22.7%)	χ^2^ = 0.98, *df* = 1, *p =* 0.32
No. history of drug dependence/abuse	1 (5.3%)	0 (0%)	χ^2^ = 1.19, *df* = 1, *p =* 0.28

Abbreviations:

GWI: Gulf War Illness.

CDC CMI: Centers or Disease Control and Prevention Chronic Multisymptom Illness.

TBI: traumatic brain injury.

PTSD: posttraumatic stress disorder.

MDD: major depressive disorder.

### 2.2 MRI image acquisition

Scanning was performed on a 3T Siemens Skyra MRI with a 20-channel head coil at the SFVAHCS. Participants were provided with earplugs and head pads to mitigate sound and head movement, and a pillow under their knees for stability. A full field of view, 1 mm isotropic, T1-weighted sagittal magnetization prepared rapid gradient echo (MPRAGE) structural scan was collected with the following parameters: TR = 2.5 s, TE = 2.98 ms, no Partial Fourier (PF), IPAT = 2, flip angle = 9°, pixel bandwidth = 240. The functional echo planar imaging (EPI) sequence had the following parameters: TR = 3s, TE = 30 ms, flip angle = 80°, no PF, IPAT = 2, pixel bandwidth = 1500, 3.312 mm in-plane and 3.3 mm through plane voxel resolution, 48 slices and 200 volumes collected across a 10-min run. Participants were instructed to keep their eyes open during the rs-fMRI scan.

### 2.3 Preprocessing and 1st level modeling

Functional preprocessing and analyses were performed with afni_proc.py (Reynolds et al., 2024) in AFNI version 24.3.10. Each participants’ anatomical T1-weighted scan was nonlinearly skull-stripped and registered to a high resolution Montreal Neurological Institute (MNI) template (Fonov et al., 2011) using sswarper2. Three main procedures were used to clean the functional data of physiological noise: First, we regressed out 6 head motion parameters and their first derivatives on per-run basis. Second, we used a head motion criterion of 0.5 mm Euclidean distance (analogous to framewise displacement) for volume censoring. Lastly, we used fast ANATICOR processing to regress out local white matter signals (Jo et al., 2020; Jo et al., 2010) from an eroded white matter mask. Functional volumes were slice-timing corrected, motion corrected to the MIN_OUTLIER volume and smoothed with a 5 mm full width at half maximum (FWHM) kernel. The head motion exclusion criterion, censoring >20% of total run volumes, resulted in the exclusion of one participant’s (a 58-year-old Air Force veteran without predicted exposure) rs-fMRI data from final analysis. All data were inspected for outliers via custom R plotting and summary statistics over quality check values.

**FIGURE 1:**
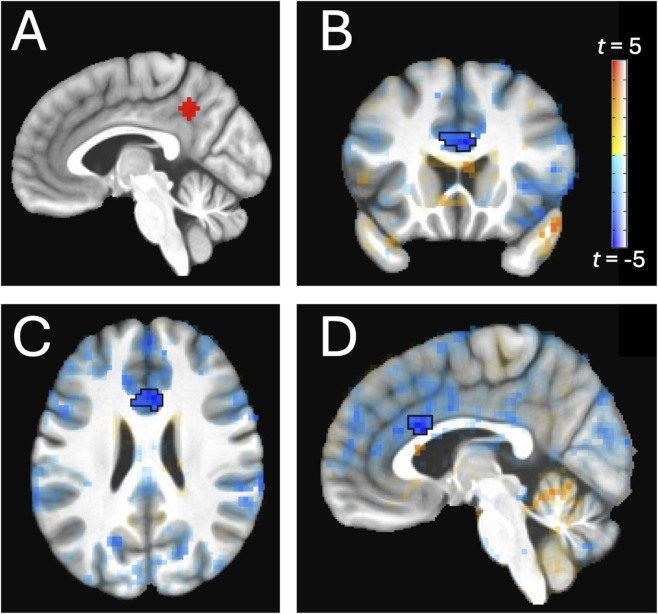
Weaker connectivity (in blue) between the left posterior cingulate cortex (PCC) seed **(A)** and the right caudal anterior cingulate cortex (ACC) in Gulf War (GW) veterans with predicated Khamisiyah exposure compared to unexposed GW veterans. Voxel map results are visualized on group-averaged anatomy with a “highlight, don’t hide” approach to the statistics (Taylor et al., 2023). The cluster outlined in black represents *p* < 0.005 voxel-level thresholding and cluster size at least 31 contiguous voxels, which constituted whole-brain correction at *p* < 0.05. **(B)** coronal view; **(C)** axial view; **(D)** sagittal view.

For each participant, residual time series were used for DMN timeseries extraction within a 6 mm spherical seed mask in the left posterior cingulate cortex (PCC, see Figure 1A; Reynolds et al., 2023) Whole-brain correlation analysis of the rs-fMRI data was conducted with the 6 mm spherical PCC seed (MNI coordinates: −5, −49, 39). For network analysis, correlations across all voxels were calculated with this timeseries and Fischer-transformed prior to group-level analyses.

### 2.4 Group level modeling and statistical correction

Voxel-level, unpaired *t*-tests of groups with and without predicted Khamisiyah exposure were modeled with 3dttest++, covarying for age and other variables of no interest (i.e., sex, service branch, CDC CMI case status, presence/absence of Kansas GWI exclusionary condition(s), current PTSD and/or MDD status, and history of TBI) at the voxel level in AFNI, testing each variable sequentially due to limited degrees of freedom. Averaged autocorrelated function (ACF) coefficients of image smoothness estimates were used for statistical multiple comparisons cluster correction (Cox et al., 2017). These averaged values were used with 3dClustSim and a 75%-thresholded averaged group mask intersected with a dilated gray matter mask for group-level cluster correction. This procedure determined that voxel-level thresholding at *p* < 0.005 with a cluster size of at least 31 contiguous voxels constituted whole-brain correction at *p* < 0.05. Voxel map results were visualized on group-averaged anatomy with a “highlight, don’t hide” approach to the statistics (Taylor et al., 2023).

### 2.5 Other measures

Other clinical and behavioral measures acquired as part of the parent have been described previously (Chao, 2018). Briefly, clinical interviews were used to assess the veterans’ histories of traumatic brain injury (TBI), lifetime psychotic or bipolar disorders, alcohol and/or substance abuse/dependence, current major depressive disorder (MDD), and current posttraumatic stress disorder (PTSD). Self-report questionnaires were used to ascertain Kansas GWI (Steele, 2000) and CDC CMI (Fukuda et al., 1998) case status. The veterans also completed a battery of neuropsychological assessments. Because we previously found evidence of inattention (i.e., more errors of omission on the CPT, Chao et al., 2011) in GW veterans with predicted exposure to the Khamisiyah plume, we present data on the participants’ performance on the Conners Continuous Performance Test Third Edition (Conners, 2014). Because we have reported evidence of hippocampal atrophy in GW veterans with predicted Khamisiyah exposure (Chao et al., 2014; Chao et al., 2017; Chao et al., 2010), we used FreeSurfer (Fischl, 2012) version 7.1 (http://surfer.nmr.mgh.harvard.edu/) to segment and parcellate the anatomical scans to derive quantitative estimates of total hippocampal volume and intracranial volume (ICV). A covariance approach (Mathalon et al., 1993) was used to adjust hippocampal volume by the FreeSurfer derived estimates of ICV with the following formula: adjusted volume = raw volume - β (ICV - mean ICV), where β is the regression coefficient when the raw volume was regressed against ICV; mean ICV reflects the group mean.

### 2.6 Statistical analyses

Group differences in demographic, military and clinical variables, and ICV were examined using Chi-squares tests for categorical variables and Welch’s t-tests for continuous variables. Group differences in cluster-extracted, Fisher-transformed functional connectivity correlation values, ICV-adjusted hippocampal volume, and CPT measures were examined with analysis of covariance (ANCOVA) that controlled for age, sex, education, current PTSD and current MDD status, history of TBI, alcohol, and drug abuse/dependence. All statistical analyses were performed with IBM SPSS Statistics version 31.

#### 2.6.1 Exploratory analyses

To test the stability of our voxel-level rs-fMRI results, we removed the subject with the second highest head motion metrics that did not exceed pre-specified criterion for censoring and we re-ran the group-level analysis without the rs-fMRI data of a 64-year-old male veteran with predicted exposure who had 17% of TRs censored from high motion.

Because the sample was predominately male and there was an imbalance in service branch between the exposure groups, we conducted exploratory analyses as a function of sex and as a function of service branch during the Gulf War, irrespective of predicted exposure status. We compared demographic and clinical variables using Chi-squares test for categorical variables and ANOVA for continuous variables. We also included sex and service branch as covariates of no interest at the voxel level in AFNI*.* Lastly, we analyzed the cluster extracted Fisher-transformed correlation values between the PCC and caudal ACC, hippocampal volume, and CPT measures as a function of sex and service branches in all veterans irrespective of predicted exposure status, and as a function of predicted exposure status among just the Army veterans.

Finally, because other GW-related co-exposures (e.g., smoke from burning oil well fires, pyridostigmine bromide, pesticide) could potentially influence DMN functional connectivity, we examined the veterans’ GW-deployment-related exposures, identified from their responses on the Kansas Gulf War Military History and Health questionnaire (Steele, 2000). Because the questionnaire queried veterans about 20 deployment-related exposures and experiences, we used Bonferroni adjustments to correct for multiple comparisons (*p* = 0.05/20 = 0.0025). Although the questionnaire also asked veterans to estimate the duration (i.e., 1-6 days, 7-30 days, 31 days or longer) of each exposure they reported, because of small cell sizes in some of the exposure/duration subgroups, we binarized the exposures as experienced or not experienced to reduce the number of comparisons.

## 3 Results

### 3.1 Study sample

Table 1 summarizes the demographic, military, and clinical characteristics of the 41 GW veterans as a function of predicted Khamisiyah exposure status. The only significant difference between veterans with and without predicted exposure was branch of military service during the Gulf War--all veterans with predicted exposure served in the Army during the Gulf War whereas only one third of the veterans without predicted exposure served in the Army during the Gulf War (χ^2^ = 18.72, *p* < 0.001). However, post-hoc exploratory analysis revealed no significant demographic or clinical differences across the service branches irrespective of exposure status. There were also no demographic or clinical differences between the two exposure groups among just the Army veterans (see Supplementary Table S1).

### 3.2 Head motion and preprocessing quality control metrics

Head motion can create spurious group findings in rs-fMRI connectivity. However, our analyses revealed no significant group differences in average head motion per TR irrespective of censoring, no group difference in averaged censored motion, and no group difference in the average number of TRs above the motion limit (see Table 2).

**TABLE 2:** Head motion and quality control (QC) metrics by predicted Khamisiyah exposure group.

Head motion and QC metrics	Exposed	Unexposed	Statistics^a^
Average motion (euclidean norm) per TR	0.1246	0.1162	*t* = −0.39, *p* = 0.70
Average (euclidean norm) censored motion	0.1137	0.1063	*t* = −0.39, *p* = 0.70
Mean number of TRs above motion threshold	3.47	2.57	*t* = −0.65, *p* = 0.52

^a^ Welch 2-sample and 2-tailed t-test.

TR, repetition time.

One veteran’s rs-fMRI data was excluded from analyses due to censoring. To test the stability of our presented results, we removed the subject with the second most-censored data (a 64-year-old male veteran with predicted exposure who had 17% of TRs censored) and re-ran the group-level analysis. The caudal ACC cluster survived the same statistical correction with only a slight reduction in the peak voxel t-statistic (from *t* = −4.59 to *t* = −4.42).

### 3.3 DMN functional connectivity

**FIGURE 2:**
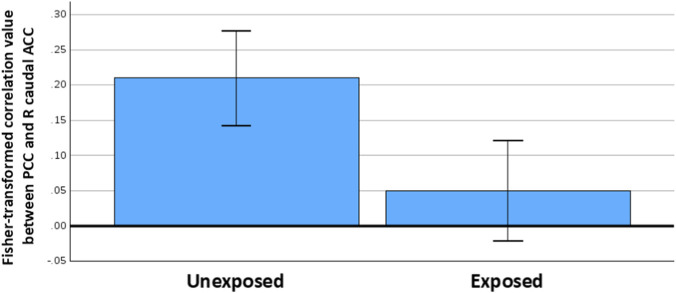
Bar graph of the extracted Fisher-transformed correlation values between the posterior cingulate seed and the right caudal anterior cingulate cluster in GW veterans with (right) and without (left) predicted Khamisiyah exposure. Values are estimated marginal means accounting for age, sex, years of education, CDC CMI case status, presence/absence of Kansas GWI exclusionary condition(s), current PTSD and/or current MDD status, histories of TBI, alcohol and/or drug abuse/dependence. Error bars represent 95% confidence interval.

Compared to unexposed GW veterans, those with predicted Khamisiyah exposure displayed weaker connectivity between the left PCC seed (−5, −49, 39) and a cluster in the right caudal anterior cingulate (ACC) (5,20,26) (see Table 3; Figures 1B-D, 2). The caudal ACC cluster remained significant (*p* < 0.005, corrected) when we included other covariates of no interest (i.e., sex, service branch, CDC CMI case status, presence/absence of Kansas GWI exclusionary condition(s), current PTSD status, current MDD status, history of TBI) at the voxel level analyses in AFNI.

**TABLE 3:** Group differences in functional connectivity to Left PCC seed.

Main effect of group	Location	# Voxels	Coordinates (x,y,z)	t-statistics
Exposed < unexposed	R Anterior cingulate	41	5,20,26	−4.59

Group difference was determined to be significant if thresholded at *p* < 0.005 with a cluster size >31 voxels.

Cluster-extracted, Fisher-transformed correlation values between the PCC and caudal ACC are reported by group in Table 4. Exploratory analyses of the cluster-extracted functional connectivity values revealed no effect of service branch or sex when all veterans were included in the analyses. The effect of predicted exposure status on the cluster-extracted, Fisher-transformed correlation values remained significant when we considered only Army veterans in the analysis (F_1,25_ = 10.05, *p* = 0.004, see Supplementary Table S2).

**TABLE 4:** Estimated marginal means^a^ of imaging and CPT measures by predicted Kahmisiyah exposure groups.

Outcome measures	Group	Estimated Marginal Mean (SD)	95% CI	Statistic	$\eta_{p}^{2}$
DMN functional connectivity^b^					
Fisher-transformed correlation value between LPCC and R caudal ACC	Exposed	0.05 (0.22)	−0.02, 0.12	F_1,39_ = 9.76^**^	0.25
	Unexposed	0.21 (0.21)	0.14, 0.28		
Hippocampal volume (in cc)					
Left HP	Exposed	4.07 (0.55)	3.89, 4.24	F_1,40_ = 4.68^*^	0.14
	Unexposed	4.34 (0.51)	4.18, 4.50		
Right HP	Exposed	4.22 (0.75)	3.98, 4.46	F_1,40_ = 2.08^†^	0.07
	Unexposed	4.46 (0.69)	4.24, 4.68		
Continuous performance test					
Number of hits^c^	Exposed	285 (10)	282, 288	F_1,40_ = 0.16	0.01
	Unexposed	286 (9)	283, 288		
Hit reaction (ms)	Exposed	430.4 (124.2)	390.8, 470.0	F_1,40_ = 0.10	0.00
	Unexposed	439.4 (114.2)	403.0, 475.8		
Response style T-score^d^	Exposed	47.9 (14.4)	43.3, 52.5	F_1,40_ = 0.40	0.01
	Unexposed	50.0 (13.3)	45.8, 54.2		
*d*’ T-score^e^	Exposed	44.4 (16.3)	39.2, 48.7	F_1,40_ = 0.02	0.00
	Unexposed	43.9 (15.0)	39.2, 49.6		
% omission T-score^f^	Exposed	47.0 (8.8)	44.1, 49.8	F_1,40_ = 0.02	0.00
	Unexposed	46.7 (8.1)	44.1, 49.3		
% commission T-score^g^	Exposed	46.2 (14.0)	41.8, 50.7	F_1,40_ = 0.02	0.00
	Unexposed	46.6 (12.9)	42.5, 50.7		
% perseveration T-score^h^	Exposed	48.5 (15.8)	43.5, 53.6	F_1,40_ = 0.00	0.00
	Unexposed	48.7 (14.5)	44.1, 53.3		
Block change T-score^i^	Exposed	52.3 (15.1)	47.5, 57.1	F_1,40_ = 0.21	0.01
	Unexposed	50.7 (13.9)	46.3, 55.1		
ISI change T-score^j^	Exposed	49.6 (16.5)	44.4, 54.9	F_1,40_ = 1.95	0.06
	Unexposed	54.9 (15.1)	50.0, 59.7		

**p=0.004, *p<0.04, ^†^p=0.16.

^a^ Estimated marginal means accounting for age, sex, education, CDC CMI, Kansas exclusionary condition(s), current PTSD, current MDD, histories of TBI, alcohol and drug abuse/dependence and (SD) reported.

^b^ data from 21 unexposed and 19 exposed GW, veterans.

^c^ Number of correctly detected targets (test has 288 targets).

^d^ Measure of how subject balanced speed vs. accuracy: scores ≥60: slow/accurate; scores ≤40: fast/inaccurate; scores 41-59: balanced.

^e^ Ability to discriminate between target and non-target stimuli; higher T score = poor discrimination ability.

^f^ Missed target stimuli.

^g^ False alarm (response to non-targets).

^h^ Inapproprate repetition of a previous response.

^i^ Slope of the change in RT, across different blocks of the test); lower scores = better performance; T scores ≥60 indicate problems with sustained attention or vigilance.

^j^ Measure of vigilance and sustained attention (higher scores = slower RTs, at longer intervals); T scores >60 indicate significant impairment.

### 3.4 Hippocampal volume

Veterans with predicted Khamisiyah exposure had smaller left hippocampal volume compared to unexposed GW veterans (F_1,40_ = 4.68, *p* = 0.039, see Table 4). Exploratory post-hoc analyses revealed no significant effect of service branch or sex on hippocampal volume irrespective of predicted exposure status (see Supplementary Table S2). There was no group difference in the Freesurfer estimated intracranial volume (ICV in veterans without predicted exposure: 1653.32 ± 173.62 cc; ICV in veterans with predicted exposure: 1665.27 ± 142.68 cc; *t* = −0.24, *df* = 39, *p* = 0.81).

### 3.5 CPT performance

There was no significant difference in CPT performance as a function of predicted exposure status. However, exploratory analyses revealed significant difference in response time to correctly detected targets (i.e., hit RT; F_3,40_ = 3.00, *p* < 0.05), d’ T-score (F_3,40_ = 3.27, *p* = 0.04), and % commission T-score (F_3,40_ = 4.08, *p* = 0.02) between the four service branches. Planned contrasts revealed marginal (*p* ≤ 0.08) differences between Air Force and Army veterans’ and Air Force and Navy veterans’ response times to correctly detected targets. Planned contrasts revealed significant (after Bonferroni adjustments) differences between Air Force and Navy veterans’ d’ T-scores (*p* = 0.029). There was also a significant difference between Air Force and Navy veterans’ % commission T-scores (*p* = 0.01). There was no effect of sex on CPT performance when all veterans were considered, irrespective of predicted exposure status, and no effect of predicted exposure status on CPT performance among just Army veterans.

### 3.6 Other GW-related exposures

Because other GW-deployment related exposure could potentially influence DMN functional connectivity, in exploratory analyses we examined the veterans’ deployment-related exposures as a function of predicted Khamisiyah exposure status. Although five exposures differed between the groups, only two (contact with prisoners of war, χ^2^ = 12.75, *p* < 0.001) and taking pyridostigmine bromide (PB) pills (χ^2^ = 9.86, *p* = 0.002) survived Bonferroni adjustments for multiple comparisons. The same two exposures were different between Army veterans with and without predicted Khamisiyah exposure; however, the *p*-values were not significant after adjustments for multiple comparisons. When we only considered veterans without predicted Khamisiyah exposure in the exploratory analyses, there were no differences in the deployment-related exposures as a function service branch (See Supplementary Table S3).

## 4 Discussion

The goal of the present study was to explore differences in DMN functional connectivity as a function of predicted exposure to the Khamisiyah plume. We found that veterans with predicted Khamisiyah exposure had weaker functional connectivity between the PCC seed and a cluster in the right caudal ACC compared to veterans without predicted exposure.

After the Gulf War ended, United Nations Security Council Special Commission (UNSCOM) inspectors confirmed the presence of sarin and cyclosarin at the ammunition storage facility in Khamisiyah, Iraq (Winkenwerder, 2002). Sarin and cyclosarin are acetylcholinesterase inhibitors, and overexposure to these chemicals can result in cholinergic overstimulation, which research has suggested may reduce DMN functional connectivity (Peeters et al., 2020; Sanda et al., 2024). However, it should be noted there is substantial uncertainty associated with the Khamisiyah plume models: First, they were conducted based on unit location during four target dates in March, 1991 and do not have specificity at the individual level (Directorate for Deployment Health Support of the Special Assistant to the Medical Readiness and Military Deployments, April 2002). Second, the United States General Accounting Office (2004) has cited a number of problems with the models, including inaccuracies in the quantity and purity of nerve agent, underestimation of explosion plume heights, under representative conditions of field tests, and wide divergence of plume patterns from the various computer plume. Although bias due to misclassification is a distinct possibility, it is difficult to determine the direction in which misclassification would have biased the results. This is because a veteran who had been exposed could have been misclassified as not exposed or *vice versa*. At minimum, misclassification would increase noise and render the true effects of exposure to the Khamisiyah plume more difficult to detect. Finally, it has been suggested that other nerve agent exposures took place during the GW (U.S. Senate Committee on Banking, Housing and Urban Affairs, 1994; Office of the Special Assistant for Gulf War Illnesses, 1996; Tucker, 1997) particularly during the early phase of the war (Haley and Tuite, 2013; Tuite and Haley, 2013). The Khamisiyah plume modeling does not include these potential earlier exposures.

Besides the plume from the demolition operations at Khamisiyah, GW veterans were exposure to other potentially hazardous chemicals during their deployment. It is possible that these other GW-related exposures contributed to the current findings of decreased DMN connectivity in veterans with predicted Khamisiyah exposure. When we assessed the veterans’ deployment-related experiences as a function of predicted exposure status, 5 GW-related experiences differed between the two exposure groups, although only two survived correction for multiple comparisons: coming into contact with prisoners of war (POW), a proxy for exposure to the organochlorine lindane that was used to delouse POWs (Sullivan et al., 2018), and taking PB pills, a prophylactic utilized by the military to protect troops against potential nerve agent exposure (Research Advisory Committee on Gulf War Veterans' Illnesses, 2008; 2014). PB is a carbamate compound that, like sarin and cyclosarin, can inhibit acetylcholinesterase. As noted earlier, overexposure to acetylcholinesterase inhibitors can result in cholinergic overstimulation, which can reduce DMN functional connectivity (Peeters et al., 2020; Sanda et al., 2024). While organochlorines such as lindane do not inhibit acetylcholinesterase, they can alter levels of glutamate and GABA receptors (Srivastava et al., 2015), which may influence the strength and stability of intrinsic connectivity within the DMN (Kapogiannis et al., 2012). It is noteworthy that chronic inflammation is one of the long-term consequence of exposure to neurotoxins such as organophosphates, carbamates, and organochlorines (Nabi and Tabassum, 2022), and there is research evidence that chronic inflammation may disrupt DMN functional connectivity, particularly between the PCC and anterior regions of the brain such as the ACC (Aruldass et al., 2021). Elevated inflammatory markers (e.g., C-reactive protein and interlukin-6) have also been associated with reduced functional connectivity between these key DMN nodes (Aruldass et al., 2021).

Although we did not detect an effect of GWI (or CDC CMI case status) on DMN functional connectivity, epidemiological research has linked the same three deployment-related exposures (nerve agents, PB, and pesticides) that differed between the veterans with and without predicted Khamisiyah exposure in the current study to GWI (Research Advisory Committee on Gulf War Veterans' Illnesses, 2008; 2014). Physiological stress (e.g., heat, cold, physical activity, sleep deprivation) associated with deployment has also been proposed to contribute to GWI symptoms (Sapolsky, 1998). Animal studies have shown that subchronic exposure to the rodent stress hormone corticosterone (CORT) can enhance the neuroinflammation associated with neurotoxicity (Kelly et al., 2012). Furthermore, prior exposure to CORT can markedly boost the neuroinflammatory effects of diisopropyl fluorophosphate (DFP), a sarin surrogate (O'Callaghan et al., 2015). This is the basis for using a combination of exposure to CORT and DFP in an animal model of GWI (O'Callaghan et al., 2015).

We replicated our previous finding of reduced hippocampal volume (in the left hemisphere) in the current sample of GW veterans with predicted Khamisiyah exposure relative to unexposed GW veterans; however, we did not detect differences in CPT performance between veterans with and without predicted exposure. This is likely because CPT measures had the smallest effect size of all the outcome measures in the current study. We had a much larger sample (64 veterans with and 64 veterans without predicted exposure) in the study where we reported significant group differences in errors of omission on the CPT (Chao et al., 2011). There is suggestive evidence that rs-fMRI is more sensitive to underlying brain changes than behavioral measures (Sheline and Raichle, 2013). For example, abnormal resting-state functional connectivity can be detected *before* structural changes manifested as cerebral atrophy; and both resting state and structural neuroimaging changes appear long before clinical symptoms manifest (Sheline and Raichle, 2013).

Although we did not observe group differences in CPT performance or clinical outcomes in this sample of 41 deployed GW veterans, other studies of individuals with nerve agent exposure have reported impairments in both cognition and mental health. As described in the introduction, Loh and colleagues (2010) described the case of a U.S. Army veteran who exhibited motor and attention deficits 8 months after he was exposed to sarin in Iraq. Cognitive (Nishiwaki et al., 2001), somatic and psychological (Sugiyama et al., 2020), and brain imaging effects (Araki et al., 2005; Yamasue et al., 2007) have been reported in survivors of the 1995 Tokyo subway sarin attack. Karimi et al. (2023) found evidence of more mental health symptoms and executive dysfunction in Iranian veterans who had been exposed to chemical weapons during the Iran-Iraq war compared to matched controls. It is worth noting that these studies have better causal certainty compared to the Khamisiyah plume models: Sarin exposure in the U.S. Army veteran described by Loh et al. (2010) was substantiated by depressed erythrocyte cholinesterase activity relative to baseline measures obtained prior to the veteran’s deployment to Iraq. Participants in the Tokyo subway sarin studies consisted of first responders and subway workers who were present at the disaster site (Nishiwaki et al., 2001) or were victims who were treated in emergency rooms for acute sarin intoxication (Araki et al., 2005; Yamasue et al., 2007). Although Karimi et al. (2023) did not describe how chemical exposure was verified in the Iranian veterans, it has previously been documented that Iran suffered hundreds of chemical attacks in the 8 years Iran–Iraq war that resulted in approximately 100,000 injuries (Hay and Roberts, 1989). In contrast, the Khamisiyah plume models predicted exposure based on the troops’ location, which carries substantial uncertainty.

As noted earlier, the Khamisiyah plume modeling does account for other nerve agent exposures that could have occurred in the Gulf War (U.S. Senate Committee on Banking, Housing and Urban Affairs, 1994; Haley and Tuite, 2013; Office of the Special Assistant for Gulf War Illnesses, 1996; Tucker, 1997; Tuite and Haley, 2013). Hearing chemical alarms sound during deployment has been used as a proxy for potential nerve agent exposure during the Gulf War (Haley et al., 2022), and we have reported associations between the self-reported frequency of hearing chemical alarms sound during GW deployment with cognitive function (Chao, 2016) and brain volume (Chao et al., 2016) in deployed GW veterans. This suggests that whatever triggered the chemical alarms during the Gulf War likely had adverse effects on the brain. In this respect, it is notable that 14 of the 22 (64%) GW veterans without predicted Khamisiyah exposure reported hearing chemical alarms sound during the Gulf War deployment, although exploratory analyses revealed no significant relationship between the frequency of hearing chemical alarms during GW deployment and DMN functional connectivity in the current study.

While there are known issues with the reliability and reproducibility of rs-fMRI data (Chen et al., 2018; Zuo et al., 2019), Mullin et al. (2025) recently reported evidence for reliable rs-fMRI results in older adults and after significant neurological compromise. The authors also concluded that global network metrics such as within-network connectivity and the default mode network were the most reliable (Mullin et al., 2025). Another study found that standard group-level rs-fMRI analyses tend to be fairly stable (Griffanti et al., 2016). Therefore, this difference in DMN functional connectivity between GW veterans with and without predicted Khamisiyah exposure warrants replication in a larger, independent cohort of deployed GW veterans. If the result remains stable after controlling for military (e.g., branch of service) and clinical (e.g., psychiatric co-morbidity) variables and convergence with objective cognitive impairment or longitudinal change, these findings will add to the literature suggesting there are persistent differences in brain function between GW veterans with and without predicted exposure to the Khamisiyah plume.

This study has some limitations that merit consideration: First, as noted earlier, the DOD/CIA Khamisiyah plume models are associated with considerable uncertainties. Second, it is possible that veterans who served with units that were in the Khamisiyah exposure areas had different deployment intensities, job roles, training, co-exposures, and post-deployment trajectories compared to the veterans who served with units were not in the exposure areas. Because we obtained pilot rs-fMRI in an *ad hoc* fashion, we did not match the participants in the two exposure groups for service branch during the Gulf War. However, post-hoc analyses revealed no demographic or clinical differences across the service branches irrespective of exposure status, and controlling for service branch in the analyses did not alter the significance of the DMN functional connectivity or hippocampal volume findings. Third, other exposures to chemical nerve agents may have occurred during the Gulf War outside of Khamisiyah, and 64% of the GW veterans without predicted exposure reported hearing chemical alarms go off during their deployed, a proxy for potential nerve agent exposure in the GW (Haley et al., 2022). Fourth, GW veterans were exposure to numerous other potentially hazardous chemicals besides chemical warfare agents. Exploratory analyses revealed differences in five deployment-related experiences between veterans with and without predicted Khamisiyah exposure, although only two exposures survived corrections for multiple comparisons. Thus, it may be possible that exposure to PB and lindane (via contact with POWs) contributed to, or may be the primary cause for, the DMN functional connectivity differences that we observed. Fifth, exposure assessment is difficult in GW studies because there are few objective military records of which soldier was exposed to which potentially hazardous substances during the Gulf War and at what levels. Thus, assessments of GW-related exposure have primarily been conducted through self-report, often years to decades after the Gulf War ended, which can be vulnerable to issues with recall bias. Finally, we cannot rule out the possibility that non-GW-related exposures and/or experiences (e.g., occupational exposures) or other deployment-related exposures that occurred after the 1991 conflict contributed to the current findings. This is because we lack information about years of military service post-Gulf War and the veterans’ exposures and experience that occurred after the Gulf War.

The cross-sectional nature of the present study precludes us from drawing conclusions about causality, and implications of the observed DMN connectivity differences between GW veterans with and without predicted Khamisiyah exposure require further study. However, there is evidence that rs-fMRI can detect brain changes in the preclinical stages of AD, and reduced functional connectivity in the precuneus/PCC region may be indicative of future cognitive decline (Chavarria-Elizondo et al., 2025; Yuan et al., 2022). Therefore, if the DMN connectivity differences between GW veterans with and without predicted Khamisiyah exposure are confirmed, it would argue for the continued investigation of the long-term consequences of predicted exposure to the Khamisiyah plume.

## Data Availability

The raw data supporting the conclusions of this article will be made available by the authors, without undue reservation.

## Supplementary material

SUPPLEMENTARY MATERIAL TABLE S1 Effects of predicted Khamisiyah exposure on default mode network resting state functional connectivity in Gulf War Veterans.
