# Supplementary material for: Effects of predicted Khamisiyah exposure on default mode network resting state functional connectivity in Gulf War Veterans
Source: Front Toxicol. 2026 Mar 5;8:1772515. doi: 10.3389/ftox.2026.1772515 (PMC12999065; doi:10.3389/ftox.2026.1772515)
Supplement: Supplementary file 1 [file Table1.docx]

**Effects of predicted Khamisiyah exposure on default mode network resting state
functional connectivity in Gulf War Veterans**

**Supplementary Table 1. Demographic and clinical characteristics by service branch**

|  | **Army** | | | **Air Force** | **Marines** | **Navy** |
| --- | --- | --- | --- | --- | --- | --- |
|  | **All** | **Exposed** | **Unexposed** |  |  |  |
| N | 26 | 19 | 7 | 5 | 6 | 4 |
| Age (years) | 56.0 (9.2) | 55.7 (8.4) | 58.7 (10.8) | 53.6 (6.2) | 50.5 (6.3) | 59.0 (5.5) |
| Education (years) | 16.1 (2.3) | 16.3 (2.6) | 15.0 (2.5) | 15.2 (3.1) | 15.3 (2.4) | 17.5 (1.9) |
| No. (%) Female | 6 (23.1%) | 4 (21.1%) | 2 (28.6%) | 1 (20%) | 0 | 0 |
| No. (%) Kansas GWI cases | 13 (50%) | 10 (52.6%) | 3 (42.9%) | 2 (40%) | 2 (33.3%) | 1 (25%) |
| No. (%) Kansas exclusionary condition(s) | 6 (23.1%) | 5 (26.3%) | 1 (14.3%) | 0 | 0 | 1 (25%) |
| No. (%) CDC CMI cases | 23 (88.5%) | 17 (89.5%) | 6 (85.7%) | 4 (80%) | 3 (50%) | 2 (50%) |
| **Military History during the Gulf War** |  |  |  |  |  |  |
| Rank  Enlisted  Officer | 20 (76.9%)  6 (23.1%) | 15 (78.9%)  4 (21.1%) | 5 (71.4%)  2 (28.6%) | 4 (80%)  1 (20%) | 5 (83.3%)  1 (16.7%) | 3 (75%)  1 (25%) |
| Component of Service  Active Duty  Reserves  National Guard | 19 (73%)  6 (23%)  1 (4%) | 14 (72.2%)  4 (22.2%)  1 (5.6%) | 5 (71.4%)  2 (28.6%)  0 | 4 (80%)  1 (20%)  0 | 6 (100%)  0  0 | 4 (100%)  0  0 |
| No. possible/probable mild-moderate TBI | 13 (50%) | 9 (47.4%) | 4 (57.1%) | 2 (40%) | 1 (16.7%) | 2 (50%) |
| No. current PTSD | 6 (23.1%) | 5 (26.3%) | 1 (14.3%) | 1 (20%) | 0 | 0 |
| No. current MDD | 5 (19.2%) | 3 (15.8%) | 2 (28.6%) | 1 (20%) | 0 | 0 |
| No. history of alcohol dependence/abuse | 9 (34.6%) | 7 (36.8%) | 2 (28.6%) | 1 (20%) | 1 (16.7%) | 1 (25%) |
| No. history of drug dependence/abuse | 1 (3.8%) | 1 (5.3%) | 0 | 0 | 0 | 0 |

Abbreviations:

GWI: Gulf War Illness

CDC CMI: Centers for Disease Control and Prevention Chronic Multisymptom Illness

TBI: Traumatic Brain Injury

PTSD: Posttraumatic Stress Disorder

MDD: Major Depressive Disorder

**Supplementary Table 2. Estimated Marginal Means^a^ (SD) of Imaging and CPT Measures by service branch**

|  | **Army** | | |  |  |  | |  |
| --- | --- | --- | --- | --- | --- | --- | --- | --- |
|  | **All**  *(n=26)* | **Exposed**  *(n=19)* | **Unexposed**  *(n=7)* | **Air Force**  *(n=5)* | **Marines**  *(n=6)* | | **Navy**  *(n=4)* | |
| **DMN functional connectivity**^b^ |  |  |  |  |  | |  | |
| Fisher-transformed correlation value of connectivity between LPCC seed and R caudal ACC | 0.09 (0.17) | 0.04 (0.22) | 0.24 (0.12) | 0.15 (0.06) | 0.25 (0.16) | | 0.21 (0.09) | |
| **Hippocampal volume (in cc)** |  |  |  |  |  | |  | |
| Left HP | 4.12 (0.48) | 4.11 (0.52) | 4.37 (0.93) | 4.32 (1.06) | 4.46 (1.11) | | 4.30 (1.23) | |
| Right HP | 4.24 (0.60) | 4.26 (0.62) | 4.46 (1.12) | 4.45 (1.33) | 4.83 (1.39) | | 4.22 (1.55) | |
| **Continuous Performance Test** |  |  |  |  |  | |  | |
| Number of Hits | 285 (8) | 285 (9) | 286 (17) | 288 (18) | 288 (20) | | 281 (20) | |
| Hit reaction (ms) | 426.5 (98.2) | 438.7 (88.5) | 393.3 (160.8) | 521.5 (206.2) | 432.0 (234.4) | | 389.2 (237.5) | |
| Response Style T-score | 48.7 (11.9) | 47.4 (12.9) | 49.8 (23.4) | 57.2 (25.0) | 44.1 (28.4) | | 48.0 (28.8) | |
| d’ T-score | 45.0 (12.7) | 45.0 (10.1) | 45.4 (18.3) | 34.9 (26.7) | 41.4 (30.4) | | 54.6 (30.8) | |
| % Omission T-score | 47.1 (7.5) | 46.9 (8.0) | 46.8 (14.5) | 47.1 (15.6) | 43.8 (17.8) | | 51.4 (18.0) | |
| % Commission T-score | 46.4 (10.6) | 47.1 (8.8) | 46.4 (16.0) | 39.0 (22.3) | 45.4 (25.4) | | 57.7 (25.7) | |
| % Perseveration T-score | 49.2 (13.4) | 48.9 (14.5) | 50.3 (26.3) | 43.9 (28.2) | 44.9 (32.1) | | 56.2 (32.5) | |
| Block change T-score | 53.2 (12.8) | 51.5 (11.2) | 51.4 (20.4) | 54.8 (26.9) | 43.1 (30.6) | | 48.5 (31.0) | |
| ISI change T-score | 50.9 (14.9) | 50.5 (13.3) | 52.8 (24.1) | 58.6 (31.3) | 53.4 (35.6) | | 53.1 (36.1) | |

^a^Estimated marginal means accounting for age, sex, education, CDC CMI, Kansas exclusionary condition(s), current PTSD, current MDD, histories of TBI, alcohol and drug abuse/dependence and (SD) reported

^b^N=40; data from one Air Force veteran censored for head motion

**Supplementary Table 3. GW-related exposures as a function of predicted exposure status and service branch**

|  | **Contact with POWs** | **Taking PB pills** | **Seeing American/Allied troops killed/badly wounded** | **Seeing Iraqi troops killed/badly wounded** | **Sleeping in a tent with fuel burning heater** |
| --- | --- | --- | --- | --- | --- |
| **Entire sample** |  |  |  |  |  |
| Exposed | **73.7%** | **94.7%** | 68.4% | 78.9% | 78.9% |
| Unexposed | **18.2%** | **50%** | 31.8% | 40.9% | 45.5% |
| χ^2^ | **12.75** | **9.86** | 5.47 | 6.07 | 4.81 |
| *p*-value | **<0.001** | **0.002** | 0.02 | 0.01 | 0.03 |
|  |  |  |  |  |  |
| **Army Veterans Only** |  |  |  |  |  |
| Exposed | 73.7% | 94.7% | 68.4% | 78.9% | 78.9% |
| Unexposed | 14.3% | 42.9% | 57.1% | 42.9% | 42.9% |
| χ^2^ | 7.39 | 8.86 | 0.29 | 3.13 | 3.13 |
| *p*-value | 0.007 | 0.003 | 0.59 | 0.08 | 0.08 |
|  |  |  |  |  |  |
| **Unexposed Veterans** |  |  |  |  |  |
| Army | 14.3% | 42.9% | 57.1% | 42.9% | 42.9% |
| Air Force | 0% | 60% | 20% | 40% | 80% |
| Marines | 50% | 83.3% | 33.3% | 66.7% | 50% |
| Navy | 0% | 0% | 0% | 0% | 0% |
| χ^2^ | 6.16 | 7.01 | 4.26 | 4.43 | 5.81 |
| *p*-value | 0.10 | 0.07 | 0.23 | 0.22 | 0.06 |

Bolded values = survived Bonferroni correction for multiple comparison
